# Molecular Investigation of Porcine Circovirus Type 3 Infection in Pigs in Namibia

**DOI:** 10.3390/pathogens10050585

**Published:** 2021-05-11

**Authors:** Umberto Molini, Giuseppe Marruchella, Frieda Matheus, Yvonne Maria Hemberger, Bernard Chiwome, Siegfried Khaiseb, Giovanni Cattoli, Giovanni Franzo

**Affiliations:** 1 School of Veterinary Medicine, Faculty of Agriculture and Natural Resources, Neudamm Campus, University of Namibia, Private Bag, 13301 Windhoek, Namibia; u.molini76@gmail.com (U.M.); magfrieda@gmail.com (F.M.); mhemberger@unam.na (Y.M.H.); bchiwome@unam.na (B.C.); 2 Central Veterinary Laboratory (CVL), 24 Goethe Street, Private Bag, 18137 Windhoek, Namibia; khaisebs@gmail.com; 3 Faculty of Veterinary Medicine, University of Teramo, Loc. Piano d’Accio S.P. 18, 64100 Teramo, Italy; 4 Animal Production and Health Laboratory, Animal Production and Health Section, Joint FAO/IAEA Division, Department of Nuclear Sciences and Applications, International Atomic Energy Agency, P.O. Box 100, 1400 Vienna, Austria; G.Cattoli@iaea.org; 5 Department of Animal Medicine, Production and Health, University of Padova, 35020 Legnaro, Italy; giovanni.franzo@unipd.it

**Keywords:** porcine circovirus type 3, pigs, PCR, Namibia

## Abstract

Porcine circovirus type 3 (PCV-3) infection is widely distributed in domestic pig populations in America, Europe, and Asia. However, no data is currently available about its presence and distribution in Africa. This study investigated the presence of PCV-3 in pigs (n = 122) in Namibia, by means of biomolecular methods. The pig samples collected (n = 122) were representative of the swine industry in Namibia, covering the major pig production facilities in the country. All of the samples tested were negative for PCV-3, and this indicated that the virus was either not present in the country or was circulating at low levels. Further studies are needed to better understand the distribution, if any, of PCV-3 in Namibia.

## 1. Introduction

The family *Circoviridae* was established in the mid-1990s and contains small (15–25 nm in diameter), non-enveloped icosahedral viruses, with a circular and single-stranded DNA genome, classified in two distinct genera: *Circovirus* and *Cyclovirus* [1,2]. Although Cycloviruses have been reported in a wide range of mammals, birds, and insects, their host range and pathogenicity are still largely unknown [3,4]. On the contrary, members of the genus *Circovirus* are known to act as relevant pathogens in vertebrates [5]. At present, four distinct circoviruses have been identified in pigs. Porcine circovirus type 1 (PCV-1) was originally discovered in 1974 as a contaminant of a porcine kidney cell line (PK-15) and, although widespread in the swine population, PCV-1 is considered non-pathogenic [5]. During the late 1990s, a second circovirus (namely, PCV-2) was identified as the primary causative agent of “porcine circovirus-associated diseases” (PCVAD), which have a large economic and welfare impact on the pig industry [6]. More recently, two additional circoviruses have been discovered in pigs (namely, PCV-3 and PCV-4). In 2016, PCV-3 was identified in the USA in sows showing PCVAD-like clinical signs and lesions, such as porcine dermatitis and nephropathy syndrome, cardiac and multisystemic inflammation [7,8]. Since then, PCV-3 has been detected in many countries and is considered widely distributed in domestic pig and wild boar populations worldwide [9]. PCV-4 was identified in pigs with severe clinical disease in the Chinese province of Hunan, but its pathogenicity is still to be proven [10]. Very little is known about the geographical distribution of PCV-4 although a recent publication has indicated that the virus is not present in Italy or Spain [11].

The present study aimed at furthering our knowledge about the geographical distribution of PCV-3 in domestic pigs in Namibia.

## 2. Results

All of the 122 tested samples, obtained from domestic pigs, were negative for PCV-3.

## 3. Discussion

Data on the presence and geographic distribution of porcine circoviruses in Africa are limited. As reviewed by Afolabi et al. [12], PCV-2 infection has been reported in domestic pigs in some sub-Saharan countries, such as South Africa, Uganda, Tanzania, and Cameroon. More recently, different PCV-2 genotypes have been detected in Namibian domestic pigs (PCV-2b and PCV-2d) and warthogs (PCV-2c) [13]. Since its original identification, a great interest raised toward the epidemiology and clinical significance of PCV-3, which has been so far identified in several countries suited to the pig production in Europe, Asia, and America [14,15]. To the best of our knowledge, no data is currently available about the presence and distribution of PCV-3 in the African continent. Based on the present study and the tested samples, there is no evidence of PCV-3 infection in Namibia. This result was unexpected given the high prevalence reported both in domestic and wild pigs suids worldwide [14,16] and considering that PCV-3 origin most likely occurred centuries ago, allowing a long-lasting viral circulation and transmission over long distances [14]. We can hypothesize that the restrictions to pig breeding due to religious practices and the effect of the African swine fever, as well as the presence of significant geographic barriers (i.e., Saharan desert) could have slowed down the spread of PCV-3 on the African continent. On the other hand, the documented presence of PCV-2, which has several biological and epidemiological similarities with PCV-3, in sub-Saharan Africa, including Namibia, goes against such a hypothesis. The sample size may also be a limiting factor in this study and does not exclude a low-level PCV-3 circulation. However, we consider this unlikely for different reasons. Firstly, the PCV-3 prevalence has been shown to be as high as 15–20%, both in domestic and wild populations [16,17,18,19]. Therefore, based on this expected prevalence, the number of samples analyzed from an estimated population of 40,000 pigs in Namibia should have allowed for the detection of low-level PCV-3 prevalence (i.e., 7%) with high (95%) confidence. This is supported by the fact that a lower number of samples (n = 88) was tested and confirmed the presence of PCV-2 in Namibia. Importantly, the current sampling was also representative of the biggest production facilities in Namibia, with approximately 40,000 pigs, located mainly in 3 districts (i.e., Mariental, Windhoek, and Tsumeb), all of which were sampled (Table 1). Of note, two of the sampled producers generate approximately 80% of the pig meat in the country. Although PCV-3 free status cannot be confidently extended to the whole Namibian pig population, it can be reasonably attributed to the main industrial production companies. The reasons for the difference compared to PCV-2 are currently unclear and will require further investigation. IfPCV-2 and PCV-3 display different susceptibilities to the implemented biosecurity measures will require further investigations. Alternatively, the circulation of highly divergent PCV-3 strains in the considered area could have affected the diagnostic assay sensitivity, in spite of three separate primer pairs being used in the sample screening. Although possible and worthy of future investigation, this hypothesis is lessened by the low evolutionary rate of PCV-3 and its low variability observed worldwide [14,15].

## 4. Materials and Methods

A total of 122 pigs were included in the present study. The minimum sample size was selected to demonstrate disease freedom in a 40,000 animal population with a confidence of 95%, considering an estimated assay sensitivity and specificity of 98% (obtained through intra-laboratory validation) and an expected prevalence of 7%. The prevalence was selected based on the results of other epidemiological studies, reporting a PCV-3 detection frequency always higher than 6% [16]. In more detail, if a random sample of at least 118 units is taken and 5 or fewer reactors are found, the probability that the population is infected at a prevalence of 7% is 0.0486.

**Figure 1:**
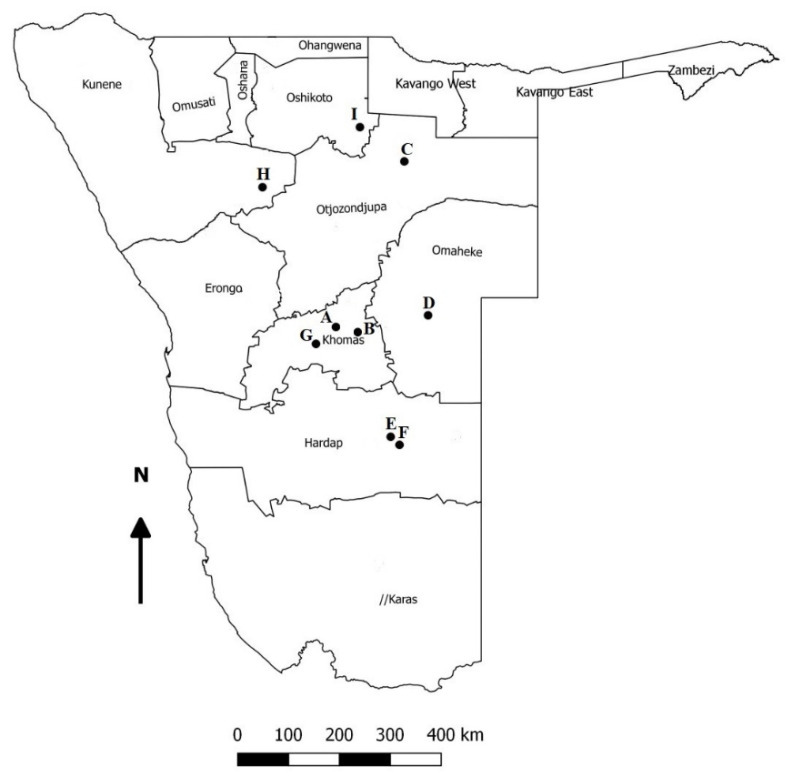
Map of Namibia showing the sampling locations as black dots.

Pigs under investigation belonged to commercial farms located in six Namibian regions (Omaheke, Oshikoto, Otjozondjupa, Hardap, Khomas, and Kunene) (Figure 1). Between 2018 and 2021, tonsils, spleen or lymph nodes were collected from slaughtered pigs (n = 70, age about 5–6 months, target weight of approximatively 75–100 kg) or from naturally dead pigs (n = 52), the latter sent to the Central Veterinary Laboratory (CVL, Windhoek, Namibia) for diagnostic purposes following an in loco post-mortem examination performed by state veterinarians. The majority of the received samples were suspicious of African swine fever. The age ranged from 3 months to 1 year old. Forty-six out of 122 pigs herein investigated had been previously tested for PCV-2 and 13 tested positive [13]. A part (50 mg) of every tissue sample was homogenized in 1 mL of sterile phosphate buffer saline (PBS) using a Tissue Lyser LT (Qiagen, Germany). Total genomic DNA was extracted from the homogenized samples using High Pure Viral Nucleic Acid Kit (Hoffman–La Roche, Switzerland) with an elution volume of 100 μL, following the manufacturer’s instructions. For each unknown sample batch, a positive (PCV-3 positive serum sample) and negative control were extracted and tested together with the diagnostic samples. For the PCV-3 specific DNA detection in the total nucleic acid extract, three pairs of primers—PCV3-1-F (forward) 5-TTACTTAGAGAACGGACTTGTAACG-3 and PCV-3-1-R (reverse) 3-AAATGAGACACAGAGCTATATTCAG-5(pair 1),PCV3-genome-1-F(forward) 5-TAGTATTACCCGGCACCTCGGAACC-3 and PCV3-genome-1-R (reverse) 3-ACAGGTAAACGCCCTCGCATGTGGG-5 (pair 2), andPCV3-genome-2-F (forward) 5-TTGCACTTGTGTACAATTATTGCG -3 and PCV3-genome-2-R (reverse) 3-ATCTTCAGGACACTCGTAGCACCAC-5 (pair 3)—were used with the following thermal profile: initial denaturation at 94 °C for 5 min and then 35 cycles of denaturation at 94 °C for 30 s, annealing at 55 °C for 30 s, and elongation at 72 °C for 60 s followed by a final elongation at 72 °C for 10 min [20]. An endogenous internal control (i.e., swine actin) was also tested for each sample in a separate reaction. A 469bp region was amplified using the primer pair Act-F: 5-AGGGTCAGGATGCCTCTCTT-3 and Act-R 5-CGGCTTCCTTTGTCCCCAATCT-3. The thermal profile was the following: initial denaturation at 94 °C for 5 min and then 35 cycles of denaturation at 94 °C for 30 s, annealing at 55 °C for 30 s, and elongation at 72 °C for 30 s followed by a final elongation at 72 °C for 5 min. All the PCRs were conducted in a reaction volume of 20 μL containing a final concentration of 10 pmol of each primer, 0.2 mM dNTPs, 1.25 mM MgCl_2_, 1× PCR buffer (QIAGEN), 2.5 U of Taq polymerase(QIAGEN), and 5 μL of template DNA. Amplification and specificity of bands were visualized using a SYBR safer stained 2% agarose gel.

## 5. Conclusions

Our data do not support the presence of PCV-3 in Namibia. We hope that the present study will stimulate further and larger investigations targeting the presence and molecular epidemiology of porcine circoviruses in Africa, which could provide useful information about the origin, evolution and relevance of porcine circoviruses in domestic and wild animal populations.

## Figures and Tables

**Table 1:** Summary of the features of the samples included in the study.

Specie	Samples	Number	Farm	Location	Region
Pig	Tissue homogenates (spleen + lymph node)	9	A	Windhoek	Khomas
Pig	Tissue homogenates (spleen + lymph node)	5	B	Windhoek	Khomas
Pig	Tissue homogenates (Lymph node)	21	C	Grootfontain	Otjozondjupa
Pig	Tissue homogenates (spleen + lymph node)	7	D	Gobabis	Omaheke
Pig	Tissue homogenates (tonsils)	15	E	Mariental	Hardap
Pig	Tissue homogenates (tonsils)	15	F	Mariental	Hardap
Pig	Tissue homogenates (tonsils)	15	G	Windhoek	Khomas
Pig	Tissue homogenates (spleen + lymph node)	10	H	Outjo	Kunene
Pig	Tissue homogenates (Lymph node)	25	I	Tsumeb	Oshikoto

## Data Availability

Not applicable.

## References

[1] Breitbart M., Delwart E., Rosario K., Segalés J., Varsani A. (2017). Ictv Report Consortium. ICTV Virus Taxonomy Profile: Circoviridae. J. Gen. Virol..

[2] Rosario K., Breitbart M., Harrach B., Segalés J., Delwart E., Biagini P., Varsani A. (2017). Revisiting the taxonomy of the family Circoviridae: Establishment of the genus Cyclovirus and removal of the genus Gyrovirus. Arch. Virol..

[3] Yan T., Li G., Zhou D., Yang X., Hu L., Cheng Z. (2020). Novel Cyclovirus Identified in Broiler Chickens with Transmissible Viral Proventriculitis in China. Front. Vet. Sci..

[4] Phan T.G., Mori D., Deng X., Rajindrajith S., Ranawaka U., Fan T.F., Bucardo-Rivera F., Orlandi P., Ahmed K., Delwart E. (2015). Small circular single stranded DNA viral genomes in unexplained cases of human encephalitis, diarrhea, and in untreated sewage. Virology.

[5] Todd D., McNulty M.S., Adair B.M., Allan G.M. (2001). Animal circoviruses. Virus Res..

[6] Segalés J., Allan G.M., Domingo M., Zimmerman J.J., Karriker L.A., Ramirez A., Schwartz K.J., Stevenson G.W., Zhang J. (2019). Circoviruses. Diseases of Swine.

[7] Palinski R., Piñeyro P., Shang P., Yuan F., Guo R., Fang Y., Byers E., Hause B.M. (2016). A Novel Porcine Circovirus Distantly Related to Known Circoviruses Is Associated with Porcine Dermatitis and Nephropathy Syndrome and Reproductive Failure. J. Virol..

[8] Phan T.G., Giannitti F., Rossow S., Marthaler D., Knutson T.P., Li L., Deng X., Resende T., Vannucci F., Delwart E. (2016). Detection of a novel circovirus PCV3 in pigs with cardiac and multi-systemic inflammation. Virol. J..

[9] Ouyang T., Niu G., Liu X., Zhang X., Zhang Y., Ren L. (2019). Recent progress on porcine circovirus type 3. Infect. Genet. Evol..

[10] Zhang H.H., Hu W.Q., Li J.Y., Liu T.N., Zhou J.Y., Opriessnig T., Xiao C.T. (2020). Novel circovirus species identified in farmed pigs designated as Porcine circovirus 4, Hunan province, China. Transbound. Emerg. Dis..

[11] Franzo G., Ruiz A., Grassi L., Sibila M., Drigo M., Segalés J. (2020). Lack of Porcine circovirus 4 Genome Detection in Pig Samples from Italy and Spain. Pathogens.

[12] Afolabi K.O., Iweriebor B.C., Okoh A.I., Obi L.C. (2017). Global Status of Porcine circovirus Type 2 and Its Associated Diseases in Sub-Saharan Africa. Adv. Virol..

[13] Molini U., Franzo G., Gous L., Moller S., Hemberger Y.M., Chiwome B., Marruchella G., Khaiseb S., Cattoli G., Dundon W.G. (2021). Three different genotypes of porcine circoviruses 2 (PCV-2) identified in pigs and warthogs in Namibia. Arch. Virol..

[14] Franzo G., He W., Correa-Fiz F., Li G., Legnardi M., Su S., Segalés J. (2019). A Shift in Porcine Circovirus 3 (PCV-3) History Paradigm: Phylodynamic Analyses Reveal an Ancient Origin and Prolonged Undetected Circulation in the Worldwide Swine Population. Adv. Sci..

[15] Franzo G., Delwart E., Fux R., Hause B., Su S., Zhou J., Segalés J. (2020). Genotyping Porcine Circovirus 3 (PCV-3) Nowadays: Does It Make Sense?. Viruses.

[16] Klaumann F., Correa-Fiz F., Franzo G., Sibila M., Núñez J.I., Segalés J. (2018). Current Knowledge on Porcine circovirus 3 (PCV-3): A Novel Virus With a Yet Unknown Impact on the Swine Industry. Front. Vet. Sci..

[17] Klaumann F., Dias-Alves A., Cabezón O., Mentaberre G., Castillo-Contreras R., López-Béjar M., Casas-Díaz E., Sibila M., Correa-Fiz F., Segalés J. (2019). Porcine circovirus 3 is highly prevalent in serum and tissues and may persistently infect wild boar (Sus scrofa scrofa). Transbound. Emerg. Dis..

[18] Franzo G., Tucciarone C.M., Drigo M., Cecchinato M., Martini M., Mondin A., Menandro M.L. (2018). First report of wild boar susceptibility to Porcine circovirus type 3: High prevalence in the Colli Euganei Regional Park (Italy) in the absence of clinical signs. Transbound. Emerg. Dis..

[19] Franzo G., Grassi L., Tucciarone C.M., Drigo M., Martini M., Pasotto D., Mondin A., Menandro M.L. (2019). A wild circulation: High presence of Porcine circovirus 3 in different mammalian wild hosts and ticks. Transbound. Emerg. Dis..

[20] Ku X., Chen F., Li P., Wang Y., Yu X., Fan S., Qian P., Wu M., He Q. (2017). Identification and genetic characterization of porcine circovirus type 3 in China. Transbound. Emerg. Dis..

